# Exploring the relationships between housing, neighbourhoods and mental wellbeing for residents of deprived areas

**DOI:** 10.1186/1471-2458-12-48

**Published:** 2012-01-18

**Authors:** Lyndal Bond, Ade Kearns, Phil Mason, Carol Tannahill, Matt Egan, Elise Whitely

**Affiliations:** 1 MRC/CSO Social & Public Health Sciences Unit, 4 Lilybank Gardens, G128RZ Glasgow, Scotland, UK; 2 Department of Urban Studies, University of Glasgow, Scotland, UK; 3 Glasgow Centre for Population Health, Glasgow, Scotland, UK

## Abstract

**Background:**

Housing-led regeneration has been shown to have limited effects on mental health. Considering housing and neighbourhoods as a psychosocial environment, regeneration may have greater impact on positive mental wellbeing than mental ill-health. This study examined the relationship between the positive mental wellbeing of residents living in deprived areas and their perceptions of their housing and neighbourhoods.

**Methods:**

A cross-sectional study of 3,911 residents in 15 deprived areas in Glasgow, Scotland. Positive mental wellbeing was measured using the Warwick-Edinburgh Mental Wellbeing Scale.

**Results:**

Using multivariate mulit-nomial logistic regressions and controlling for socio-demographic characteristics and physical health status, we found that several aspects of people's residential psychosocial environments were strongly associated with higher mental wellbeing. Mental wellbeing was higher when respondents considered the following: their neighbourhood had very good aesthetic qualities (RRR 3.3, 95% CI 1.9, 5.8); their home and neighbourhood represented personal progress (RRR 3.2 95% CI 2.2, 4.8; RRR 2.6, 95% CI 1.8, 3.7, respectively); their home had a very good external appearance (RRR 2.6, 95% CI 1.3, 5.1) and a very good front door (both an aesthetic and a security/control item) (RRR 2.1, 95% CI 1.2, 3.8); and when satisfaction with their landlord was very high (RRR 2.3, 95% CI 2.2,4.8). Perception of poor neighbourhood aesthetic quality was associated with lower wellbeing (RRR 0.4, 95% CI 0.3, 0.5).

**Conclusions:**

This study has shown that for people living in deprived areas, the quality and aesthetics of housing and neighbourhoods are associated with mental wellbeing, but so too are feelings of respect, status and progress that may be derived from how places are created, serviced and talked about by those who live there. The implication for regeneration activities undertaken to improve housing and neighbourhoods is that it is not just the delivery of improved housing that is important for mental wellbeing, but also the quality and manner of delivery.

## Background

It is well established that physical and mental health are related to the physical and built environment [1-4]. In general, poor people live in deprived areas and experience poorer health [1]. While we know that physical and mental health are related to the physical and built environment, less is known about which particular features of the built environment influence either physical or mental health [5,6].

Furthermore, housing and neighbourhoods are not just defined by their physical aspects but can also be considered as a psychosocial environment, similarly to the way they are considered in studies of health inequalities and the workplace [7]. That is, neighbourhoods can be environments that promote a person's positive or negative experience or view of themselves in relation to others, for example in terms of trust, control, self-esteem and status. We argue that these psychosocial mechanisms make plausible a hypothesised association between perceived housing and neighbourhood characteristics and positive mental health.

To date, however, cross-sectional, longitudinal and intervention studies that explore associations between mental health and residential circumstances have mostly used measures of mental illness, with little evidence relating to more positive mental wellbeing [8-12]. Mental wellbeing is not the absence of mental illness [13], and it can be argued that whilst only minorities of the population are mentally ill, mental wellbeing is something that everyone experiences, albeit to varying degrees. Recognising this, there is therefore a need to measure it appropriately in order to assess its relationship with housing and neighbourhood characteristics.

In this paper we focus on some of the poorest communities in Glasgow (which also have multiple health problems) to better understand what aspects of residents' perceptions of their housing and neighbourhoods are associated with their mental wellbeing. The high-level aim is to inform targeted individual- and area-based strategies, including urban regeneration programmes that attempt to improve residents' mental health and quality of life in disadvantaged areas.

### Relationship between mental health and the physical and built environment

People living in deprived areas have poorer mental health than those in less deprived areas [14,15], and researchers have explored the residential factors that might influence this inequality. A number of studies have examined the relationship between mental health and the built environment and neighbourhoods (e.g. [10,11,16]). Characteristics of the built environment found to be associated with poor mental health include housing type (e.g. high-rise housing), poor housing quality and the internal environment (damp, warmth etc.), crowding and neighbourhood noise [10,16]. The quality of the neighbourhood with respect to the physical attributes of the environment (e.g. derelict buildings, green space etc.), perceived neighbourhood problems (e.g. fear of crime), and limited opportunities for social participation have all been associated with poor mental health [10-12,16,17].

Changes in mental health problems over time have not been shown to be strongly related to residential neighbourhoods. For example, a study of mental health outcomes over a ten-year period showed that, whilst mental health scores (using GHQ-12, a measure of minor psychiatric morbidity) were worse on average for social renters living in the most deprived areas, there was no effect of area deprivation on trajectories of mental health over the study period [9].

Studies which have examined the effect on mental health of housing improvements or housing-led regeneration have shown some positive impacts on mental health, although the evidence is stronger for targeted housing improvement [18-21]. Those examining warmth and energy efficiency improvements have reported significant improvements in vitality and happiness [18] but mixed results for depression, anxiety or mental illness [20]. The evidence of effects on mental health of area-based housing improvement programmes is more mixed [20]. For example, a controlled study of mental health outcomes (using GHQ-12) in a regeneration area in Manchester showed no improvement and possibly some negative effects upon mental health (reporting mental distress) due to the regeneration programme [22,23], while another UK study reported a significant reduction in self-reported anxiety or depression [24].

### Mental wellbeing not mental ill-health

A limitation of these studies, we argue, has been the way mental health has been assessed. Most studies examining the effects of housing and neighbourhood on mental health have used measures of mental illness (depression, anxiety and stress being the most common) (e.g. [8-12]). Only one study that we are aware of used a measure of mental health rather than mental illness [25]. That study used questions from the SF12 asking about happiness and vitality.

There are two issues with using mental ill health measures in assessing positive mental health in the general population. Firstly, relatively few people in the general population have symptoms of mental illness, thus scoring low or zero on these measures. It is therefore arguable whether such instruments are 'fit for the purpose of establishing ...[ways]... to improve mental health at the general population level' [26] and questionable whether they are sensitive enough to the impacts of changes in the residential environment.

Secondly, and perhaps more important, is the recognition that mental health or wellbeing is not equivalent to the absence of mental illness. The WHO has defined mental health as: 'a state of wellbeing in which every individual realises his or her own potential, can cope with the normal stresses of life, can work productively and fruitfully, and is able to make a contribution to her or his community.'[13]. Positive mental health or wellbeing can therefore be conceptualised as a state of health, happiness and prospering, comprising two dimensions, namely how we feel (the subjective experience of affect and life satisfaction) and how we function (psychological functioning, good relationships with others, and self-realisation) [27-29]. It is perhaps clearer to see how this conceptualisation of positive mental health or wellbeing might be more closely related to the psychosocial environment, and therefore amenable to changes in that environment. More specifically, we can consider the residential domain of housing and the neighbourhood to constitute an important psychosocial environment which affects our feelings and our functioning in relation to others.

### Public policy, regeneration and mental wellbeing

Reducing health inequalities is a key policy priority for both the UK and Scottish governments with both 'mental illness and mental wellbeing [being] specific priorities...' [30]. The need for a focus on promotion of positive mental health has been recognised and is on the agenda in a number of policy areas beyond that of health, including social justice, social inclusion, education etc. [26]. In Scotland the promotion of positive mental wellbeing is considered to be integral to health improvement [26,30]. One of the Scottish Government's 45 performance measures for the period 2008-11 is 'to increase the average score of adults on the WEMWBS scale by 2011' [31].

Improving mental wellbeing, and reducing inequalities in mental wellbeing, is also a stated aim of regeneration [32]. Regeneration commonly includes a number of dimensions or approaches to improving deprived communities: e.g. (1) upgrading the physical housing and neighbourhood environments; (2) partly through (1) generating a new (or renewed) psychological dynamic among the resident group--to 'lift their spirits' and aspirations; and (3) building capacity to enhance individual and collective capabilities and opportunities so that people can achieve more. In relation to the first two of these dimensions, it is clear that an understanding of which modifiable environmental characteristics are strongly associated with mental wellbeing could help inform regeneration planning and implementation in order to help achieve overall public policy health objectives. Hence, there is a rationale in the first instance for assessing the strength of these associations.

This article, therefore, looks at the associations between aspects of housing and neighbourhoods and mental wellbeing to see whether higher mental wellbeing *might *be expected as an outcome of regeneration activities, and, if so, which aspects of residential circumstances appear most likely to have an impact in that they seem strongly associated with levels of mental wellbeing. In all cases, we are looking at residents' perceptions of their residential environments rather than independent measures of them, since mental wellbeing is about a psychological dynamic, i.e. whether it is possible to change people's general state of mind/wellbeing, through changing how they assess their residential circumstances. Furthermore, the focus is on perceptions measured at the level of individuals.

Ultimately, the aim of this paper is to examine the associations of individually perceived aspects of housing and neighbourhoods with the mental wellbeing of residents in deprived areas, after adjusting for individual characteristics.

Specifically, we:

• examine whether mental wellbeing scores for residents of deprived areas are associated with residents' perceptions of aspects of housing and neighbourhoods upon which regeneration *might act*;

• estimate the relative strength of those associations so as to identify the relative importance of different aspects of the residential environment for mental wellbeing

Using the Warwick-Edinburgh Mental Wellbeing Scale (WEMWBS), a comprehensive measure of wellbeing [27,33], we consider three types of residential circumstances as potential influences upon mental wellbeing. Each of these involve judgements by occupants, made, in our view, partly in relation to other people and what is considered 'normal', or a reasonable expectation, within society:

• Residential circumstances and psychosocial benefits

• Dwelling type and perceived housing quality

• Perceived neighbourhood quality

## Methods

This paper draws on data from the second cross-sectional survey of GoWell, a long-term study of 15 deprived communities in Glasgow undergoing major housing investment and area regeneration over a 10-15 year period. All but one of the 15 study areas is in the 15% most deprived areas in Scotland on the Scottish Index of Multiple Deprivation, with six of the areas in the very worst, or most deprived, 5% (see [34] for more details about the GoWell study design and methods). The GoWell areas are broadly representative of deprived areas in Glasgow [35].

### Sampling and recruitment

The 15 areas varied in population size and to obtain sufficiently powered samples for each area we used a mixed sampling strategy (involving a census in six areas and random stratified cross-sectional sampling in nine areas). We selected households in each study area from the addresses in the most recent version of the Royal Mail Postal Address File within the postcode units that define the study areas. Where selected homes had more than one householder, only the householder with the most recent birthday was interviewed (i.e. one householder was interviewed per selected household). Households selected for cross-sectional surveys were posted information sheets and letters inviting them to take part. Study information leaflets were produced in English, Arabic, Urdu, Cantonese and Turkish. Fieldworkers made up to five attempts to contact selected homes in person to seek consent to participate.

A face-to-face questionnaire lasting around 40 min was verbally administered by fieldworkers at participants' homes with responses recorded using CAPI (Computer Assisted Personal Interviewing). Interpreters were available or assistance from other household members obtained if the interviewee did not speak English (or one of several of the languages common amongst UK non-English speakers in which some of the contracted fieldworkers were also fluent). The achieved sample of householders was 4,657, with an overall response rate of 47.5%, which is good for a survey of deprived area populations.

Ethics approval for the study was given by the NHS Scotland B MREC (no. 05/MRE10/89).

### Measures

The outcome of interest here is mental wellbeing. To consider how regeneration might impact upon wellbeing, the other independent variables are organised into groupings related to residential circumstances and psychosocial factors, and perceptions of residents' house and neighbourhood.

### Mental wellbeing

Mental wellbeing was assessed using the Warwick-Edinburgh Mental Wellbeing Scale (WEMWBS) [27,33]. The scale has good psychometric properties [36]. In the validation of WEMWBS, Tennant et al. [27] examined the relationships between other measures of positive mental health (e.g. WHO-5, SPWB, PANAS) and mental ill health (GHQ- 12, PANAS). They reported relatively high correlations with the other positive wellbeing measures (correlations ≥ 0.7) and moderate, negative correlations with the GHQ- 12 (r = -0.53) measure of mental ill health. They concluded that 'respondents scoring the same on the GHQ-12 had a range of WEMWBS scores, so although lower WEMWBS scores tend to be associated with higher GHQ-12 scores, one is not simply the inverse of the other. The two scales are therefore not measuring the same thing' [27].

The scale has 14 items covering: positive affect (feelings of optimism, cheerfulness, relaxation); positive functioning (energy, clear thinking, self-acceptance, personal development, competence and authority); and relationships with others. Respondents are asked to what extent they have been feeling that way over the past two weeks. Responses are summed, with higher scores indicating greater wellbeing. Internal consistency of the scale in this study was high (Cronbach alpha 0.92). Low, average and high wellbeing in the current analyses were defined as: < 1 standard deviation below the population mean in Scotland (50.7), +/-1 standard deviation (9.9) around the mean, and > 1 standard deviation above the mean, respectively. We chose to trichotomise the WEMWBS scores to better show the associations with average and high wellbeing, rather than the effect of a one-point increase in the continuous wellbeing score.

### Socio-demographic factors

We included the following socio-demographic factors in our analysis: gender, ethnicity, age, household type (young adults, single-parent family, two-parent family, or household with two or more adults over 64 years of age), and educational attainment. Economic status was assessed using three variables: employment status, income source, and regular access to a private vehicle.

### Health

In this analysis we used the first item of the SF-12v2 Health Survey [37]--a self-reported assessment of health from poor to excellent--and a question about whether the respondent considered themselves to have a long-standing illness or disability.

### Residential circumstances and psychosocial benefits

Here we include housing tenure and the length of time the respondent had lived in their home and neighbourhood, as well as their perceptions of the internal and external reputation of the neighbourhood, whether their neighbourhood had changed for better or worse in the previous two years, satisfaction with their home, neighbourhood and landlord, and whether their home or neighbourhood made them feel they were doing well in their life.

Tenure can be considered to convey material and psychosocial advantages [38]. Housing tenure is included here (rather than as a socio-demographic factor), because for our study group, in deprived areas, we believe tenure has greater importance as a psychosocial factor than as a structural or material factor. Whilst we recognise that housing tenure, in particular ownership, can confer financial advantages, for marginal home-owner groups it often results in financial disadvantage and psychological stress [39].

Furthermore, and perhaps more importantly, the preference for private consumption, possession and ownership of goods--especially housing--has been explained by sociologists as a 'cultural phenomenon rather than something inherent to the objects themselves', providing benefits of identity, autonomy and ontological security [[40], p.328-9]. Thus, housing is not simply economic consumption but also 'symbolic consumption' enabling the external communication of identity [41]. This has assumed ever greater importance as home ownership has become 'normalized' in British society and discourse as a positional good which conveys status, normal aspiration, and notions of being a good citizen and parent [42].

Length of residence is also included here as it serves as a proxy for place attachment, the two having been shown to have a strong relationship in recent research in the UK [43]. Place attachment itself confers psychological benefits for residents by giving lives meaning, value and significance and contributing to identity and self-esteem [44,45].

### Dwelling type and perceived housing quality

Residents provided information about type of residence (house, high- or low-rise flat, etc.), the existence of internal and external problems, access to a garden, perception of overall condition of the residence, and specifically about insulation, the external appearance and repair, and the condition of their front door. They were also asked about their satisfaction with any improvements that had been made to their residence. We have included dwelling type here for similar reasons to those mentioned above in relation to housing tenure. Dwelling type also contributes to psychosocial benefits pertaining to identity, status and self-esteem, with strong general preferences for houses over flats, and for larger properties with more rooms and additional space over and above smaller properties [46]. This is reflected in the strong emotional content involved in the house marketing and selection process [47].

### Perceived neighbourhood quality

Residents were asked whether the following environmental incivilities in their local neighbourhood were a serious or a slight problem, or not a problem: vandalism, graffiti, other deliberate damage to property or vehicles; abandoned or burnt-out cars; rubbish or litter lying around; vacant or derelict buildings and sites; and untidy gardens. The number of serious problems was summed and then coded as: 3 or more serious incivilities, 1-2 serious incivilities, or no serious incivilities.

Quality of local amenities (play areas; schools; youth and leisure services; shops; banking or financial services; childcare or nurseries; health centre or GP service) was assessed. The number of amenities assessed as 'poor' or 'very poor' was counted and coded as 3 or more, 1 or 2, or none.

Residents were asked to rate the attractiveness of the buildings and environment in their neighbourhood and whether it was quiet and peaceful. Neighbourhood quality was based on the number of 'fairly' or 'very poor' responses to questions about attractive buildings, attractive environment, and a quiet and peaceful environment.

### Method of analysis

Multinomial logistic regression was used to assess the associations between housing and neighbourhood environment and average or high wellbeing compared with low wellbeing, adjusting for individual socio-demographic factors, and taking account of clustering within GoWell areas. STATA Version 9 was used for all analyses [48].

## Results

From the sample of 4,657 complete data were available from 3,911 households for this study. Forty-three percent of respondents were male. Table 1 presents the socio-demographic, economic and general health data for the sample, for those with low, average and high wellbeing.

**Table 1:** Mental Wellbeing by Sociodemographic Factors

	Sample demographics		Wellbeing		
	
	Total		Low	Average	High
	**N**	**%**	**%**	**%**	**%**

**Gender**					

Male	1,986	42.7	36.7	32.1	31.2

Female	2,671	57.3	39.3	34.0	26.7

**Age or respondent**					

16-24	348	7.6	25.9	28.2	46.0

25-39	1,318	28.8	33.2	34.5	32.3

40-54	1,240	27.1	39.9	31.8	28.3

55-64	635	13.9	40.9	35.1	23.9

65+	1,039	22.7	44.4	34.2	21.5

**Ethnic group**					

British--white	3,626	78.9	38.6	33.9	27.5

Non-British	1,022	21.1	36.8	30.4	32.8

**Highest educational attainment**					

None	2,689	57.7	45.0	31.7	23.4

Completed compulsory schooling	883	18.9	30.1	36.4	33.5

Post compulsory secondary school	290	6.2	27.9	30.7	41.4

Post school further training	578	12.4	29.2	36.0	34.8

University qualification	217	4.66	24.4	35.5	40.1

**Household structure**					

Young adult	2,186	5.1	37.8	32.7	29.5

Single-parent family	807	17.3	38.8	35.6	25.6

Two-parent family	661	14.2	29.1	32.5	38.4

Older person	1,003	21.5	44.4	33.2	22.4

**SF12 General health**					

Excellent	854	18.3	26.4	29.5	44.2

Very good	1,049	22.5	20.8	37.9	41.4

Good	1,316	28.3	32.6	39.4	28.0

Fair/Poor	1,438	30.8	63.1	26.3	10.6

**Long-standing illness**					

Yes	1,409	30.3	60.8	26.5	12.6

**Employment status**					

Working	1,427	30.7	19.3	39.0	41.7

Not working	1,935	41.7	46.7	28.4	24.9

Retired	1,281	27.6	42.9	35.4	21.7

**Source of income/benefits**					

State benefits	2,377	51.1	45.5	30.2	24.2

Private (income from employment)	1,007	21.6	17.6	37.5	44.9

Rather not say/don't know	1,273	27.4	40.8	35.3	23.9

**Vehicle ownership**					

Yes	1,203	25.8	23.8	36.9	39.3

* Inc 64 stated don't know

Multivariate analyses of the socio-demographic (including economic and health) factors are shown in Table 2. This table presents relative risk ratios (RRR) and 95% confidence intervals (95% CIs) and compares those with average and high mental wellbeing with those reporting low wellbeing. All variables except gender and age were strongly associated with mental wellbeing. The strongest associations were with good general health, with those in good healths being twice or four times as likely to report average or high wellbeing, respectively. Conversely, having a long-standing illness was associated with a reduced likelihood of reporting average or high levels of wellbeing. After health, the resource-related variables (employment, income source and car ownership) had the biggest effect on wellbeing, followed then by the demographic variables (ethnicity, education and household type). All subsequent analyses were adjusted for all socio-demographic variables.

**Table 2:** Relative Risk Ratio (RRR) for Average and High Mental Wellbeing by Sociodemographic Factors

	Average vs. low wellbeing	High vs. low wellbeing	*p*-value
	**RR (95% CI)**	**RR (95% CI)**	

**Gender**			

Male	1	1	

Female	0.96 (0.86, 1.07)	0.87 (0.71, 1.05)	*.22*

**Age or respondent**			

16-39	1	1	

40-64	0.99 (0.79, 1.25)	0.88 (0.68, 1.13)	*.54*

65+	0.93 (0.62, 1.39)	0.69 (0.37, 1.30)	

**Ethnic group**			

British--white	1	1	

Non British	0.72 (0.58, 0.89)	0.78 (0.54, 1.13)	*.01*

**Highest educational attainment**			

None	1	1	

Compulsory schooling	1.46 (1.13, 1.89)	1.68 (1.15, 2.46)	

Post compulsory secondary school	1.07 (0.74, 1.54)	1.53 (1.05, 2.25)	

Post school further training	1.34 (0.98, 1.84)	1.47 (1.01, 2.14)	

University qualification	1.38 (0.99, 1.93)	1.52 (1.09, 2.13)	*< .001*

**Household structure**			

Young adult	1	1	

Single-parent family	1.06 (0.81, 1.39)	0.74 (0.53, 1.04)	

Two-parent family	0.98 (0.75, 1.28)	1.01 (0.75, 1.36)	

Older adult	0.90 (0.65, 1.26)	1.25 (0.72, 2.15)	*.03*

**SF12 General health**			

Fair/poor	1	1	

Excellent/very good/good	2.21 (1.82, 2.67)	4.42 (3.49, 5.62)	*< .001*

**Long-standing illness**	0.55 (0.44, 0.68)	0.39 (0.32, 0.48)	*< .001*

**Employment status**			

Not working	1	1	

Working	1.46 (1.20, 1.78)	1.49 (1.33, 1.67)	

Retired	1.91 (1.53, 2.39)	1.46 (1.07, 2.00)	*< .001*

**Source of income/benefits**			

State benefits	1	1	

Private	1.61 (1.18, 2.19)	1.52 (1.08, 2.16)	*< .001*

Rather not say/don't know	0.90 (0.64, 1.27)	0.57 (0.33, 1.00)	

**Vehicle ownership**			

No	1	1	

Yes	1.56 (1.22, 1.99)	1.86 (1.32, 2.62)	*< .001*

* Each variable is adjusted for all others in the table

### Residential circumstances and psychosocial benefits

The associations between residential factors and mental wellbeing are shown in Table 3. Length of residence in the home and area, poor external area reputation and intention to move in the next 12 months were not markedly associated with mental wellbeing after adjustment for socio-demographic, economic and physical health measures. When all of these variables were included simultaneously in the model, the strongest associations were with satisfaction with the landlord, and perceptions of personal progress (my home and my area make me feel I am doing well in my life); in all cases, highly positive views more than doubled the likelihood that someone would also report high wellbeing. Also significant, though with smaller effects, were perceiving that one's neighbours thought highly of the area (internal reputation), and being very satisfied with one's home.

**Table 3:** Relative Risk Ratio (RRR) for Average and High Mental Wellbeing by Residential Psychosocial Benefits

	Adjusted for confounders*			Reciprocally adjusted**		
	
	Average vs. low	High vs. low	*p*-value	Average vs. low	High vs. low	*p*-value
**Tenure**						

Rented	1	1		1	1	

Owner occupied	1.49 (1.19, 1.88)	1.49 (1.04, 2.14)	*0.001*	1.33 (1.06, 1.67)	1.10 (0.71, 1.70)	*0.04*

**Length of residence in home**				Not in model		

< 2 years	1	1				

3-5 years	1.06 (0.90, 1.25)	0.96 (0.74, 1.23)				

6-10 years	0.85 (0.74, 0.98)	0.90 (0.68, 1.19)				

11-20 years	0.90 (0.77, 1.06)	0.81 (0.61, 1.08)				

21+ years	0.82 (0.64, 1.06)	0.76 (0.58, 1.01)	*0.55*			

**Length of residence in area**				Not in model		

< 2 years	1	1				

3-5 years	1.20 (0.99, 1.44)	1.00 (0.75, 1.33)				

6-10 years	0.94 (0.72, 1.24)	0.96 (0.67, 1.40)				

11-20 years	0.94 (0.76, 1.16)	0.93 (0.70, 1.22)				

21+ years	0.97 (0.78, 1.22)	0.99 (0.72, 1.36)	*0.74*			

**Good internal area reputation**						

Disagree	1	1		1	1	

Neither	1.70 (1.38, 2.09)	1.60 (0.93, 2.74)		1.45 (1.18, 1.78)	1.41 (0.85, 2.32)	

Agree	2.38 (1.84, 1.05)	2.87 (1.81, 4.55)	*< 0.001*	1.55 (1.26, 1.92)	1.57 (1.01, 2.43)	*0.001*

**Poor external area reputation**				Not in model		

Agree	1	1				

Neither	1.07 (0.88, 1.29)	0.97 (0.72, 1.32)				

Disagree	0.81 (0.58, 1.11)	0.89 (0.65, 1.22)	*0.08*			

**Neighbourhood change**						

Worse	1	1		1	1	

Same	1.45 (1.17, 1.79)	1.65 (1.13, 2.40)		1.04 (0.79, 1.36)	1.06 (0.75, 1.50)	

Better	1.33 (1.04, 1.71)	2.01 (1.40, 2.89)		0.94 (0.70, 1.26)	1.20 (0.88, 1.65)	

Don't know	0.95 (0.69, 1.30)	1.37 (1.01, 1.85)	*< 0.001*	0.77 (0.54, 1.09)	1.03 (0.80, 1.33)	*0.09*

**Satisfaction with home**						

Not satisfied	1	1		1	1	

Fairly satisfied	1.51 (1.26, 1.82)	1.31 (1.04, 1.65)		0.99 (0.81, 1.22)	0.81 (0.66, 1.00)	

Very satisfied	1.61 (1.34, 1.93)	3.39 (2.61, 4.41)	*< 0.001*	0.95 (0.79, 1.15)	1.27 (0.99, 1.62)	*< 0.001*

**Satisfaction with neighbourhood**						

Not satisfied	1	1		1	1	

Fairly satisfied	1.66 (1.43, 1.93)	1.48 (1.04, 2.10)		0.99 (0.77, 1.27)	0.85 (0.58, 1.27)	

Very satisfied	1.56 (1.19, 2.04)	2.80 (2.02, 3.88)	*< 0.001*	0.86 (0.62, 1.21)	0.92 (0.63, 1.33)	*0.26*

**Satisfaction with landlord/factor**						

Not satisfied	1	1		1	1	

Fairly satisfied	1.70 (1.34, 2.15)	1.47 (1.09, 1.97)		1.39 (1.12, 1.73)	1.23 (0.98, 1.56)	

Very satisfied	1.62 (1.10, 2.38)	4.52 (2.87, 7.12)	*< 0.001*	1.28 (0.85, 1.92)	2.32 (1.49, 3.61)	*< 0.001*

**My home makes me feel I am doing well**						

Don't agree	1	1		1	1	

Agree	1.90 (1.54, 2.34)	2.18 (1.64, 2.91)		1.30 (1.02, 1.66)	1.57 (1.12, 2.19)	

Strongly agree	2.39 (1.80, 3.17)	7.12 (5.05, 10.03)	*< 0.001*	1.58 (1.15, 2.16)	3.21 (2.15, 4.80)	*< 0.001*

**Living in this area makes me feel I am doing well**						

Don't agree	1	1		1	1	

Agree	2.50 (2.03, 3.08)	2.63 (2.00, 3.46)		1.89 (1.49, 2.38)	1.87 (1.45, 2.41)	

Strongly agree	2.55 (1.94, 3.33)	7.56 (5.99, 9.55)	*< 0.001*	1.88 (1.28, 2.76)	2.56 (1.76, 3.72)	*< 0.001*

* Adjusted for socio-demographic variables

** Adjusted for socio-demographic variables and all other psychosocial factors

### Dwelling type and perceived housing quality

Table 4 presents the associations between positive mental wellbeing and housing variables, adjusting for human and economic factors and general physical health. Access to a garden was no longer statistically significant after reciprocal adjustment in the full model. The strongest housing effects, after mutual adjustment, were related to the external appearance of the home and the front door (both an aesthetic and a security- or control-related item): highly positive views of both these items more than doubled the likelihood of high wellbeing. Good insulation (a warmth and comfort issue) was the next most important dwelling item.

**Table 4:** Relative Risk Ratio (RRR) for Average and High Mental Wellbeing by Dwelling Type and Perceived Housing Quality

	Adjusted for confounders*			Reciprocally adjusted**		
	
	Average vs. low	High vs. low	*p*-value	Average vs. low	High vs. low	*p*-value
**House type**						

High-rise flat	1	1		1	1	

Low-rise flat	1.13 (0.86, 1.49)	1.04 (0.69, 1.56)		0.96 (0.77, 1.19)	0.86 (0.56, 1.31)	

4-in-a-block	1.58 (0.95, 2.62)	1.74 (0.79, 3.84)		1.26 (0.76, 2.08)	1.17 (0.48, 2.83)	

House	1.99 (1.50, 2.65)	1.84 (1.07, 3.16)	*< 0.001*	1.56 (1.23, 1.98)	1.02 (0.55, 1.90)	*0.03*

**Number of internal problems**						

None	1	1		1	1	

1 or 2	0.80 (0.62, 1.03)	0.86 (0.59, 1.25)		0.94 (0.72, 1.23)	1.24 (0.83, 1.86)	

3+	0.41 (0.28, 0.59)	0.40 (0.29, 0.56)	*< 0.001*	0.69 (0.50, 0.95)	0.84 (0.56, 1.26)	*0.01*

**Number of external problems**						

None	1	1		1	1	

One	0.77 (0.59, 1.02)	0.58 (0.42, 0.81)		0.97 (0.76, 1.23)	1.01 (0.70, 1.45)	

2+	0.37 (0.25, 0.54)	0.35 (0.25, 0.50)	*< 0.001*	0.45 (0.28, 0.72)	0.73 (0.34, 1.57)	*0.02*

**Home improvements**						

No	1	1		1	1	

Yes (< very sat)	0.99 (0.79, 1.24)	0.52 (0.41, 0.64)		1.12 (0.90, 1.38)	0.67 (0.55, 0.81)	

Yes (very sat)	1.02 (0.76, 1.37)	1.10 (0.83, 1.46)	*< 0.001*	1.07 (0.80, 1.44)	0.90 (0.66, 1.22)	*< 0.001*

**Access to garden**						

No garden	1	1		1	1	

Shared garden	1.55 (1.14, 2.12)	1.36 (0.78, 2.37)		1.20 (0.93, 1.56)	1.15 (0.60, 2.19)	

Own garden	1.56 (1.22, 2.00)	1.64 (1.07, 2.50)	*< 0.001*	0.97 (0.74, 1.26)	1.03 (0.67, 1.56)	*0.67*

**Insulation**						

Poor	1	1		1	1	

Neither	1.54 (1.16, 2.05)	1.26 (0.86, 1.84)		0.97 (0.74, 1.27)	0.94 (0.60, 1.46)	

Fairly good	2.46 (1.68, 3.61)	1.98 (1.28, 3.05)		1.10 (0.81, 1.48)	1.07 (0.68, 1.68)	

Very good	2.51 (1.69, 3.72)	5.20 (3.17, 8.54)	*< 0.001*	1.02 (0.73, 1.43)	1.54 (0.96, 2.47)	*0.01*

**External repair**						

Poor	1	1		1	1	

Neither	0.85 (0.65, 1.13)	0.66 (0.49, 0.89)		0.46 (0.37, 0.59)	0.39 (0.27, 0.57)	

Fairly good	1.73 (1.21, 2.47)	1.25 (0.86, 1.82)		0.69 (0.53, 0.89)	0.51 (0.35, 0.76)	

Very good	2.14 (1.50, 3.06)	3.86 (2.42, 6.16)	*< 0.001*	0.85 (0.60, 1.20)	0.87 (0.51, 1.48)	*< 0.001*

**External appearance**						

Poor	1	1		1	1	

Neither	1.49 (1.16, 1.92)	1.34 (1.02, 1.76)		0.95 (0.70, 1.28)	1.35 (0.86, 2.11)	

Fairly good	2.09 (1.50, 2.90)	2.37 (1.70, 3.30)		0.91 (0.63, 1.33)	1.73 (1.05, 2.85)	

Very good	2.36 (1.71, 3.25)	7.01 (4.94, 9.95)	*< 0.001*	0.90 (0.55, 1.49)	2.62 (1.33, 5.14)	*< 0.001*

**Front door**						

Poor	1	1		1	1	

Neither	1.38 (0.99, 1.93)	1.10 (0.67, 1.78)		0.96 (0.61, 1.50)	1.00 (0.53, 1.87)	

Fairly good	2.46 (1.55, 3.89)	2.60 (1.55, 4.38)		1.38 (0.88, 2.15)	1.84 (1.01, 3.34)	

Very good	2.23 (1.45, 3.41)	5.03 (2.96, 8.54)	*< 0.001*	1.24 (0.83, 1.85)	2.10 (1.16, 3.77)	*< 0.001*

* Adjusted for socio-demographic variables

** Adjusted for socio-demographic variables and for perceived housing quality

### Perceived neighbourhood quality

Table 5 presents the associations between residents' perceptions of neighbourhood environmental characteristics and wellbeing. Controlling for human, economic and health factors, environmental incivilities, poor neighbourhood quality and poor quality amenities and services were all associated with lower wellbeing. Neighbourhood environmental quality remained independently and significantly associated with wellbeing after mutual adjustment, as were all three component elements thereof (Table 6). The strongest effect was for the attractiveness of the local environment (other than buildings), where a highly positive rating trebled the likelihood of high wellbeing.

**Table 5:** Relative Risk Ratio (RRR) for Average and High Mental Wellbeing by Perceived Neighbourhood Quality

	Adjusted for confounders*			Reciprocally adjusted**		
	
	Average vs. low	High vs. low	*p*-value	Average vs. low	High vs. low	*p*-value
**Incivilities (number of serious problems)**						

None	1	1		1	1	

1 or 2	0.83 (0.71, 0.97)	0.82 (0.62, 1.08)		0.97 (0.81, 1.15)	0.95 (0.72, 1.25)	

3+	0.64 (0.53, 0.77)	0.68 (0.46, 1.02)	*< 0.001*	0.95 (0.75, 1.19)	1.01 (0.67, 1.51)	*0.95*

**Neighbourhood Environment: attractiveness**						

None	1	1		1	1	

One	0.64 (0.52, 0.79)	0.64 (0.47, 0.88)		0.66 (0.53, 0.83)	0.64 (0.46, 0.90)	

2 or 3	0.38 (0.34, 0.43)	0.39 (0.29, 0.53)	*< 0.001*	0.40 (0.34, 0.48)	0.39 (0.30, 0.50)	*< 0.001*

**Quality of amenities/services (poor or very poor)**						

None	1	1		1	1	

1 or 2	0.82 (0.66, 1.02)	0.84 (0.64, 1.11)		0.91 (0.73, 1.12)	0.93 (0.72, 1.20)	

3+	0.69 (0.59, 0.80)	0.74 (0.50, 1.10)	*0.002*	0.90 (0.73, 1.10)	0.98 (0.65, 1.48)	*0.76*

* Adjusted for socio-demographic variables

** Adjusted for socio-demographic variables and for perceived neighbourhood quality

**Table 6:** Relative Risk Ratio (RRR) for Average and High Mental Wellbeing by Perceived Neighbourhood Environment

	Adjusted for confounders*			Reciprocally adjusted**		
	
	Average vs. low	High vs. low	*p*-value	Average vs. low	High vs. low	*p*-value
**Attractiveness of buildings**						

Poor	1	1		1	1	

Neither	1.44 (1.21, 1.71)	1.06 (0.81, 1.40)		1.14 (0.88, 1.46)	0.81 (0.63, 1.05)	

Fairly good	2.59 (2.24, 3.00)	2.17 (1.60, 2.94)		1.52 (1.11, 2.09)	1.07 (0.82, 1.39)	

Very good	3.15 (2.25, 4.41)	6.28 (4.25, 9.30)	*< 0.001*	1.98 (1.22, 3.20)	1.93 (1.35, 2.76)	*< 0.001*

**Attractiveness of environment**						

Poor	1	1		1	1	

Neither	1.40 (1.19, 1.65)	1.21 (0.86, 1.69)		1.03 (0.78, 1.35)	1.19 (0.84, 1.68)	

Fairly good	2.77 (2.31, 3.31)	2.79 (1.99, 3.92)		1.63 (1.19, 2.22)	2.12 (1.47, 3.06)	

Very good	2.71 (1.95, 3.77)	6.94 (4.50, 10.71)	*< 0.001*	1.44 (0.94, 2.20)	3.29 (1.86, 5.82)	*< 0.001*

**Quiet and peaceful environment**						

Poor	1	1		1	1	

Neither	1.65 (1.34, 2.04)	1.15 (0.81, 1.63)		1.44 (1.15, 1.80)	1.06 (0.75, 1.50)	

Fairly good	2.63 (2.09, 3.32)	2.21 (1.55, 3.14)		1.71 (1.35, 2.17)	1.41 (1.03, 1.94)	

Very good	2.42 (1.87, 3.14)	4.15 (2.61, 6.59)	*< 0.001*	1.39 (1.08, 1.78)	1.72 (1.03, 2.86)	*< 0.001*

* Adjusted for socio-demographic variables

** Adjusted for socio-demographic variables and for perceived neighbourhood quality

## Discussion

This study has examined the associations between housing, neighbourhood and mental wellbeing after controlling for personal characteristics (age, gender, household type and economic factors) and found that the residential and environmental aspects of people's houses and neighbourhoods were significantly associated with positive mental wellbeing. In particular, for residential aspects, perceiving the area as having a good internal reputation, being satisfied with house and landlord and feeling that both the home and neighbourhood contribute to a sense of doing well were all associated with average or higher than average levels of mental wellbeing. In terms of environmental aspects, average and high levels of wellbeing were associated with living in a house (rather than a flat), having a home in good repair, living in an area perceived as having attractive buildings, and living in an attractive, quiet and peaceful environment.

Our findings are generally in agreement with studies examining the associations between the built environment and mental health [16,49]. The perceived quality of the neighbourhood has previously been found to be associated with mental health as measured by GHQ-12 [17], although the association between the presence or absence of amenities or services and mental health has not been examined in previous studies. In our study we found the quality of neighbourhood amenities was related to mental wellbeing at the univariate level but *not *once environmental quality was included in the model. Depression has previously been associated with the built form (deck access flats, and post-1969 buildings) and disused buildings but not with open spaces, graffiti or access to a private garden [11].

In his review, Evans [16] proposed that '...unresponsive landlords' (p 538) might explain the association between the built environment and mental (ill) health, although he provided no studies or data to support this explanation. Our study not only provides support for such an explanation but also show there is a strong association between mental wellbeing and satisfaction with the landlord. No previous studies have reported the association between mental wellbeing and internal reputation of the area and feelings of progress.

Overall, our study confirms that there is merit in considering the residential domain as a psychosocial environment that affects residents' mental wellbeing. The most important aspects of the residential domain for mental wellbeing for those people living in deprived areas were not found to be housing tenure or houses as a built form (aspects that policy for disadvantaged communities has often focused on), but rather a set of psychosocial factors which are all relational in nature: satisfaction with one's landlord (relational to a service provider); a sense of personal progress derived from one's home and neighbourhood (relational to a prior state and trajectory); and the internal reputation of one's neighbourhood (relational to how other nearby people talk about one's place). Several aesthetic aspects of the home and neighbourhood were also found to be significantly associated with mental wellbeing (e.g. external appearance of the home; attractiveness of the local environment), and these may also be considered as potentially relational and psychosocial since they are concerned with appearance and its role in attributed status, helping to make people feel good about themselves and their position in society. With the exception of general health, these psychosocial factors had stronger associations with mental wellbeing than most of the socio-demographic and resource-based variables, as might be expected.

This study contributes to a greater understanding of how perceptions of neighbourhood might contribute to mental wellbeing. Importantly, we found that not only are the quality and aesthetics of housing and neighbourhoods associated with mental wellbeing, but so, too, are feelings of respect, status and progress derived from how places are created, serviced and talked about by those who live there. The residential domain is both an important source and signifier of one's position in society in terms of relative position and advancement, self-esteem and self-efficacy. These are precisely those things also found to be important in respect of other psychosocial environments such as the workplace [7,50].

### Strengths and limitations

The cross-sectional design of this study precludes attribution of cause. While the associations we have found between mental wellbeing and many of the aspects of residential and environmental capital are strong, we cannot yet conclude that the built or psychosocial environment contributes to positive wellbeing. Most of the outcomes are self-reported and therefore subject to bias related to common method variance. It might also be hypothesised that some self-reported outcomes might be more susceptible to common method variance than others (e.g. perhaps self-reported psychosocial outcomes are more susceptible to participants general mental state compared to self-reported social status or physical environment measures--but we do not have the evidence to test this theory). Unfortunately, using more objective measures, usually proposed as a way of avoiding this problem, is not appropriate for many of the constructs or factors we are interested in, these being residents' perceptions of their environment. Our planned longitudinal study of residents staying in their original areas or moving to other parts of the city will help give a clearer indication of whether mental wellbeing scores are responsive to changes in residential circumstances. Another useful approach, which we have not been able to adopt here, may have been to measure personality types to account for the fact that different kinds of people may respond in different ways to unfavourable circumstances, with some people adjusting their expectations and evaluations to better accord with the reality they face [24].

This study, on the other hand, has many strengths. It is a large study with a relatively good response rate (~50%) for a survey of this type in deprived areas. It examined a large number of housing and neighbourhood factors and used an appropriate measure of mental wellbeing.

## Conclusion

### Implications for public policy and regeneration

According to philosophers and sociologists, the rise of meritocracy and the growing importance of status in society [51] has led to a situation whereby the importance of places of residence lies partly (or largely) in the way they affirm an individual's sense of identity and social position; the meaning of place is judged in a relational sense [52]. What is important about where you live is how it makes you feel about yourself in relation to wider society. This understanding of places is reflected in our findings of the strong associations between mental wellbeing of the physical and service qualities of housing and neighbourhoods, for example issues of appearance, relations with local institutions (the most important in deprived areas being landlord and council), sharing a positive valuing of the area with one's neighbours, and having a home and neighbourhood that people would aspire to.

Scottish regeneration policy is said to be about people and place [32], with ministers aiming 'to tackle not just the physical needs but the economic and social regeneration of the area'[53], including empowering communities to be able to work together and make things happen for themselves [31]. English regeneration policy has similarly emphasised both people and place dimensions, prioritising the reduction of worklessness and 'fostering ambition' so that people take advantage of opportunities, but also emphasising the desirability of improving the attractiveness of places and of targeting services to 'underperforming areas'[54]. Mental wellbeing constitutes an important objective for regeneration and is relevant to other goals pertaining to empowerment, aspirations and employment. Our work illustrates the strong connections between these dimensions of regeneration. We have found that the quality and aesthetics of housing and neighbourhoods are associated with mental wellbeing, but so, too, are feelings of respect, status and progress which may be derived from how places are created, serviced and talked about by those who live there.

## Competing interests

The authors declare that they have no competing interests.

## Authors' contributions

LB, AK, CT, PM and ME contributed to the GoWell study and participated in its design and coordination. EW undertook the statistical analysis and contributed to the interpretation of the results. All contributed to drafting the manuscript and read and approved the final manuscript.

## Pre-publication history

The pre-publication history for this paper can be accessed here:

http://www.biomedcentral.com/1471-2458/12/48/prepub
